# Career shift phenomenon among doctors in tacloban city, philippines: lessons for retention of health workers in developing countries

**DOI:** 10.1186/1447-056X-10-13

**Published:** 2011-10-06

**Authors:** Meredith P Labarda

**Affiliations:** 1 School of Health Sciences, University of the Philippines-Manila in Palo, Leyte, the Philippines

**Keywords:** Career-shift, doctors, nurses, immigration/emigration/migration, retention, health workers, job satisfaction/dissatisfaction

## Abstract

**Background:**

At the height of the global demand for nurses in the 1990s, a phenomenon of grave concern arose. A significant number of medical doctors in the Philippines shifted careers in order to seek work as nurses overseas. The obvious implications of such a trend require inquiry as to the reasons for it; hence, this cross-sectional study. The data in the study compared factors such as personal circumstances, job satisfaction/dissatisfaction, perceived benefits versus costs of the alternative job, and the role of social networks/linkages among doctors classified as career *shifters *and *non-shifters*.

**Methodology:**

A combined qualitative and quantitative method was utilized in the study. Data gathered came from sixty medical doctors practicing in three major hospitals in Tacloban City, Philippines, and from a special nursing school also located in the same city. Respondents were chosen through a non-probability sampling, specifically through a chain referral sampling owing to the controversial nature of the research. A set of pre-set criteria was used to qualify doctors as *shifters *and *non-shifters*. Cross-tabulation was carried out to highlight the differences between the two groups. Finally, the Wilcoxon-Mann-Whitney test was utilized to assess if these differences were significant.

**Results:**

Among the different factors investigated, results of the study indicated that the level of job satisfaction or dissatisfaction and certain socio-demographic factors such as age, length of medical practice, and having children to support, were significantly different among shifters and non-shifters at p ≤ 0.05. This suggested that such factors had a bearing on the intention to shift to a nursing career among physicians.

**Conclusion:**

Taken in the context of the medical profession, it was the level of job satisfaction/dissatisfaction that was the immediate antecedent in the intention to shift careers among medical doctors. Personal factors, specifically age, support of children, and the length of medical practice gained explanatory power when they were linked to job satisfaction or dissatisfaction. On the other hand, factors such as perceived benefits and costs of the alternative job and the impact of social networks did not differ between shifters and non-shifters. It would then indicate that efforts to address the issue of physician retention need to go beyond economic incentives and deal with other sources of satisfaction or dissatisfaction among practicing physicians. Since this was an exploratory study in a particular locale in central Philippines, similar studies in other parts of the country need to be done to gain better understanding of this phenomenon at a national level.

## Background

The United Nations Millennium Declaration in 2000, articulated in terms of eight (8) development goals, sought to achieve a more equitable world order by 2015. However, many of its indicators, especially the attainment of health equity, are in danger of missing their target. For instance, in the Philippines, many health authorities have conceded that the goal of achieving improved maternal health through reduction in the maternal mortality ratio of 52 deaths per hundred thousand live births, is unattainable. The lack of access to health services and competent health workers largely contributes to the problem. This is further compounded by the continued emigration of medical personnel, thus, complicating efforts to achieve health equity in developing countries.

Migration of Philippine health workers is not a new phenomenon. It began in 1950s and was initially considered a stopgap measure to diffuse tight local employment markets. But as the overseas demand for health workers increased markedly, it became institutionalized, in order to gain hard currency for the government through the inflow of remittances. The present policy is based on the premise that migration eases domestic unemployment, brings in substantial dollar remittances, and increases productivity by enabling skill transfer [1]. This has, in turn, encouraged massive deployment of human health resources from the Philippines, in the past three decades. In 1975 there were only 36, 035 overseas workers recorded but this increased to nearly a million by the year 2000 [2].

The contribution of overseas workers in shoring up the Philippine economy is generally lauded to the point of considering them as modern-day heroes. In the past few years, however, public outcry and worries about the state of the Philippine health care system has been triggered by the phenomenon of doctors retraining to become nurses in the hope of securing employment overseas [3]. At the height of the global demand for nurses, doctors were retraining as nurses at a rate of 1, 200 per year, with at least 9, 000 doctors becoming MDs-RNs or "nursing-medics" [4]. To date, at least 6, 000 doctors have emigrated to the United States as nurses. Moreover, in 2003-2005 more than 4, 000 physicians took the Philippine Board of Nursing Licensure Examinations. Around 3, 000 doctors were enrolled in more than 45 nursing schools offering customized courses for physicians all over the country during this period [4]. Recent World Health Organization figures show that there are 90, 370 physicians in the country making a density of 12 doctors per 10, 000 of the population [5]. In other words, when one compares these figures, more than ten per cent (10%) of the physician workforce have shifted to nursing careers or were actively in pursuit of it at the height of the demand for nurses overseas. What probably has, so far, saved the health infrastructure from collapse (due to this "hemorrhage" of qualified medical practitioners) is the global recession that has temporarily contracted labor markets and closed many traditional recipient countries from hiring foreign workers, including those from the Philippines.

## Objectives

The objective of this study was to explore the factors affecting the exit of physicians to seek work abroad as nurses. Specifically, doctors who were planning to work abroad as nurses were compared with doctors who planned to practice within the country, using the following criteria:

1) socio-demographic and career profiles;

2) level of job satisfaction;

3) sources of job satisfaction and job dissatisfaction;

4) perceived benefits and costs of alternative job; and

5) social network/linkages

Central to this issue, then, is to understand what has pushed medical doctors to consider leaving their practice in lieu of other work opportunities. A review of the literature on job changing posits economic needs as the primary determinant of this move [6-9]. However, in the case of Filipino doctors moving to other countries as nurses, there is a paucity of studies explaining what factors are at play when one decides to shift from this occupation to one traditionally considered as lower in occupational ranking [10]. While not negating the role that economics plays in the career-shift process, *this paper looks beyond the allure of financial reward and security and explores other factors at play when highly skilled doctors shift careers to become nurses abroad*. Bringing these to light should help in some measure, in (1) understanding this career shift phenomenon; and (2) finding a durable solution to the problem of physician attrition in the country. Hopefully, then, it may lead to better retention strategies for our physician workforce and, perhaps, contribute to the attainment of the Millennium Declaration development goals.

## Methodology

This cross-sectional study used semi-structured questionnaires to gather information during the second quarter of 2007. Tacloban City was chosen as study site since there were undocumented reports that as many as half of all doctors practicing in the city and nearby localities were training as nurses with plans of going overseas. Tacloban City is the capital of Eastern Visayas, a region with one of the highest maternal mortality ratios in the Philippines.

Due to the controversial nature of the research, respondents were chosen through non-probability sampling, specifically through chain referral sampling. A set of inclusion criteria was then used to identify thirty (30) respondents as "shifters" and another thirty (30) as "non-shifters". For the *shifters*, the researcher started with a doctor in one of the hospitals in Tacloban City, who was known to have finished a nursing degree after medical school and was willing to be part of the study. This respondent, in turn, gave names of other doctors she personally knew who had enrolled in nursing programs in order to work overseas. This led to the identification of respondents for inclusion in the research which, then, included consultants and resident physicians in three major hospitals in Tacloban City and doctors from various health facilities in nearby localities of Leyte enrolled in a special nursing program designed for physicians. As for the *non-shifters*, the researcher asked for doctors in the three major hospitals in Tacloban City and nearby health facilities who were also willing to be part of the study. Descriptive statistics were generated. Cross-tabulation was done to highlight the differences between shifters and non-shifters. The Wilcoxon-Mann-Whitney test was utilized to assess if these differences were significant.

## Results

### Demographic profile

In terms of demographic parameters, a significant difference (at p ≤ 0.05) was noted between shifters and non-shifters only with regards to *mean age *and *having *or *not having children to support*. Shifters were relatively older (mean age of 42) compared to the non-shifters (mean age of 33). While both shifters and non-shifters had an average of two (2) children, the majority of shifters had children (70%) compared to non-shifters (40%). The rest of the demographic variables were not significantly different. Table 1 shows the socio-demographic profile for both shifters and non-shifters.

**Table 1:** Socio-demographic profile of respondents

Socio-demographic Profile	Shifters N = 30	Per cent	Non-shifters N = 30	Per cent	Total N = 60	*p*-value
Gender						0.793
Male	13	43	12	40	25	
Female	17	57	18	60	35	
**Total**	30		30		60	
**Civil status**						0.133
Single	15	50	8	27	23	
Married	15	50	19	63	34	
Widowed	0	0	2	7	2	
Separated	0	0	1	3	1	
**Total**	30		30		60	
**Spouse occupation**						0.514
**Unemployed**	2	13	3	16	5	
Physician	6	40	5	26	11	
Government Employee	3	20	2	10	5	
Nurse	1	7	1	5	2	
Engineer	0	0	2	10	2	
Others	3	20	6	32	9	
**Total**	15		19			
**Children**						0.02
Without children	18	60	9	30	27	
With children	12	40	21	70	33	
**Total**	30		30		60	
Mean number of children	2		2			0.2
Mean age	33		42			0.049

### Career profile

Comparing shifters and non-shifters in terms of career profile, only *the number of years of medical practice *and *years since graduating from medical school *were found to be significantly different. Generally, career shifters had graduated from medical school earlier than non-shifters. Thus the length of medical practice for shifters was more than twice (14 years on the average) than that of non-shifters (6 years on the average).

In the majority of both shifters (57%) and non-shifters (67%), the preparatory course for Medicine (M.D.) was Bachelor of Science in Biology. The primary work setting for 77% of non-shifters and 50% of the shifters was in a private hospital. In both groups, 20% worked in a government hospital. Of the shifters, 13% came from Rural Health Units (RHUs) while only 3% of non-shifters worked in the RHUs.

All *shifters *in the study were either pursuing a Nursing degree or had already obtained it. From the data gathered, 47% of the shifters already had a Nursing degree while the rest expected to obtain it between the years 2007 to 2010. It can be noted, too, that almost all of the shifters (97%) already had an intention to leave the medical profession soon. In fact, 33% of the shifters intended to leave within the year of study, another 33% in the following 3 to 5 years, and 30% within 1 to 2 years, while only 3% of shifters had no specific timetable. Their target country of work was usually the United States of America (USA) (90%), while the rest identified the United Kingdom (7%) and Australia (3%). Some shifters had already sat for the licensure examinations for a foreign country. Specifically, 37% had undertaken the Commission on Graduates of Foreign Nursing Schools (CGFNS) examinations and 33% the National Council Licensure Examination (NCLEX) examination. In addition, 37% of the shifters had already applied for nursing employment abroad, either directly or through an agency.

### Sources of job satisfaction and dissatisfaction

Literature on the subject has identified various intrinsic aspects of the medical profession that contribute to *motivation *factors as sources of satisfaction and several extrinsic components that comprise the *hygiene *factors as sources of dissatisfaction [11]. These sources of satisfaction (intrinsic factors) and dissatisfaction (extrinsic factors), identified by, both, shifters and non-shifters, are enumerated in Table 2.

**Table 2:** Sources of Satisfaction (Motivation Factors) and Dissatisfaction (Hygiene Factors)

	Level of Job Satisfaction (Mean Score)		Wilcoxon-Mann- Whitney Test (z value)
Aspects of Job	Shifters	Non-shifters	
***Motivation factors***			
How work can benefit other people	3.27	3.37	-0.591
Opportunities to develop new skills	2.50	2.93	-2.810***
Opportunities for advancement	2.23	2.77	-2.943***
Level of responsibility	2.83	2.87	-0.057
Growth of your medical practice	2.43	2.90	-2.923***
Work gives you a sense of personal accomplishment	2.80	3.10	-1.646*
Receive appropriate recognition for your work	2.63	2.77	-0.823
Value of your job	2.90	3.17	-1.348
Opportunities to acquire advance training	2.23	2.90	-4.216***
Total Motivation Factors	23.83	26.77	-3.406***
***Hygiene Factors***			
Salary/income	2.20	2.57	-1.536*
Working hours	2.63	2.60	-0.544
Physical working environment	2.53	2.67	-0.304
Relationship with fellow doctors/ medical staff	3.20	3.23	-0.113
Workload	2.50	2.63	-0.556
Balance between your professional time and personal/family time	2.47	2.60	-0.997
Resources available to support work	2.43	2.87	-2.312**
Health policies and laws	2.03	2.60	-2.715***
Overall job security	2.20	2.77	-3.211***
**Total Hygiene Factors**	22.20	24.53	-1.923**
TOTAL	46.03	51.30	-2.548***

Note: *** Significant at *p *= .01; ** Significant at *p *= .05; * Significant at *p *= .10

Respondents also identified aspects of the medical profession that they perceived to be favorable. These are shown in Table 3. The results indicate that a majority of shifters perceived a sense of fulfillment/personal satisfaction in treating the sick as the most favorable aspect of their profession. For some, satisfaction came not only from seeing people healed but also from the intellectual challenge the profession offers. Other favorable aspects included the actual practice of required and acquired skills/specialized knowledge and social recognition. People's high regard for the medical profession and the social prestige that goes with it, was personally satisfying for some.

**Table 3:** Perceived Favorable and Unfavorable Aspects of the Medical Profession

	Respondent classification	
	Shifter	Non-shifter
FAVORABLE ASPECTS*		
Sense of fulfillment/personal satisfaction	20	23
Social value and prestige	2	7
Social recognition	9	6
Widened social contact/interaction	3	6
Economic compensation	1	1
Challenging nature of the profession	6	2
Practice of required and acquired knowledge/skills in the profession	12	6
Opportunities for career growth	0	2
Others	2	3
UNFAVORABLE ASPECTS*		
Low salary/less work benefits	18	12
Heavy/tiring workload	10	5
Long working hours/excessive time commitment	13	17
Heavy responsibility/accountability	1	4
Stress/demands of the profession	7	5
Rules and policies in the workplace/country	2	2
Overall job security	4	1
Lack of medical resources/facilities	1	2
Political instability in the country	2	0
Others	2	7

Note: *Count is based on multiple responses

Like the shifters, a majority of the *non-shifters *also perceived a sense of fulfillment/personal satisfaction in helping others and treating the sick as the most favorable aspect of their profession. Other favorable aspects for non-shifters included the social value and prestige of the medical profession, social recognition, as well as wider social contacts and interaction. Finally, the practice of specific knowledge/skills of the medical profession was also a source of satisfaction.

The respondents also identified some aspects of their profession that they perceived to be unfavorable. A majority of the shifters perceived low salary/less work benefits as the most unfavorable aspect of their profession. Other unfavorable aspects included long working hours/excessive time commitment and a heavy/tiring work load. For many doctors medical practice was seen as a highly demanding profession that sometimes pushes one to neglect one's family. Moreover, the heavy burden of committing fatal mistakes also weighed heavily on the minds of many of the respondent physicians.

On the other hand, for the majority of non-shifters, it was also the long working hours and the excessive time commitment that was perceived to be the most unfavorable aspect of their profession. Other unfavorable aspects ranked low salary/less work benefits, heavy/tiring workload, and stress/demands of the profession, among others (See Table 3). Added to these were the economic problems of many patients that interfered with ideal medical practice management.

### Levels of satisfaction and dissatisfaction

Statistical analysis was done using the Wilcoxon-Mann-Whitney test in order to test if the differences between the mean scores of the two groups, in terms sources of satisfaction and dissatisfaction were significant . A higher score in motivation factors signified greater satisfaction, while a higher score in hygiene factors signified less dissatisfaction. The results revealed that there were significant differences in the mean scores for motivation factors at *p *= .01 (*z *value = -3.406) and hygiene factors at *p *= .05 (*z *value = -1.923) between shifters and non-shifters.

In terms of satisfaction as measured by *motivation factors*, shifters scored lower (mean score of 23.83) when compared to non-shifters (mean score of 26.77). Moreover, in terms of *hygiene factors*, shifters were more dissatisfied (means score of 22.20) than non-shifters (mean score of 24.53). When motivation and hygiene factors were combined, it also showed that a significant difference existed at *p *= .05 (*z *value = -2.548). This meant that when motivation and hygiene scores were taken together, non-shifters were generally more satisfied with their jobs than shifters as shown in Table 4.

**Table 4:** Mean score for motivation and hygiene Factors as levels of satisfaction/dissatisfaction

Factors affecting levels of job satisfaction	Level of Job Satisfaction (Mean Score)		Wilcoxon-Mann-Whitney Test (one-tailed)
	Shifters	Non-Shifters	z value
**Motivation Factors**	23.83	26.77	-3.406***
**Hygiene Factors**	22.20	24.53	-1.923**
All Factors	**46.03**	**51.30**	-2.548***

Note: *** Significant at *p *= .01; ** Significant at *p *= .05; * Significant at *p *= .10

The statistical test results for each specific aspect of the medical profession revealed that a significant difference existed in terms of some enumerated motivation factors (*p *= 0.05). Shifters were less satisfied than non-shifters with the following sources: opportunities to develop new skills, opportunities for advancement, growth of medical practice, and opportunities to acquire advance training.

In terms of sources of dissatisfaction, the test statistics also showed a significant difference between shifters and non-shifters in terms of the following: health policies/laws, overall job security, and resources available to support work (*p *= 0.05). Shifters were more dissatisfied with these specific hygiene factors compared to non-shifters.

### Perceived benefits and costs of shifting to a nursing career

The respondents perceived a number of factors as benefits of shifting to a nursing career. Most notably, the majority of both shifters and non-shifters saw higher income as one of the benefits of such a shift. Other perceived benefits for both groups included more opportunities for migration, better working conditions abroad, and greater chances for career advancement.

On the other hand, respondents also considered some of the costs of shifting to a nursing career. The majority of shifters perceived lower status, loss of opportunity to practice medicine, along with loss of autonomy and independence, as disadvantages of the career shift. Meanwhile, for a majority of non-shifters, the loss of opportunity to practice as physicians, and separation from family and friends were seen as disadvantages of shifting to a nursing career.

### Social network/Linkages

Shifters and non-shifters alike were noted to have linkages with other doctors who had shifted to a nursing career abroad. In fact, all the shifters and all but one of the non-shifters personally knew of other doctors who had shifted to a nursing career. All the respondents knew of doctors working as nurses in the USA, while some were acquainted with doctors working as nurses in the United Kingdom, Saudi Arabia, Canada, Ireland and in the United Arab Emirates. In a majority for both groups, these doctors were their friends, relatives, classmates, former colleagues at work, or acquaintances.

It can be further noted that more than half of the shifters indicated that their linkages with these doctors had influenced their intention to shift to a nursing career. A third of the non-shifters, although they had no intentions of shifting to a nursing career at the time of the interview, claimed that these networks did make them consider shifting to a career in nursing as well. Specifically, they were encouraged by the feedback of better compensation and working conditions abroad, while a few were encouraged by the feedback of a better life/family life abroad. Encouraging feedback from social networks notwithstanding, the study found that sixty-six per cent (66%) of the non-shifters did not even think of shifting to a nursing career.

Both shifters and non-shifters had a wide array of other social relationships, which included people whom they consulted for career shift intentions. In fact, all but one shifter consulted other people about their intention to shift to a nursing career. Results showed that for shifters, the three (3) most influential people they consulted for the nursing career shift were their spouses (mean rank of 5.6), followed by their colleagues (5.0), and parents (4.7). Non-shifters on the other hand, identified their parents as having the most influence on their career decisions (mean rank of 8.9), followed by siblings, and colleagues. It can also be noted that the social networks of a majority of the shifters approved of their intention to shift careers and assisted the shifters in various ways. Specifically, these social networks offered words of support and encouragement, some provided helpful information about employment opportunities abroad, while others provided referrals to job placement agencies.

## Discussion

### Differential variables affecting career shift process among medical doctors

#### Socio-demographic factors

Among the different factors investigated, the study indicates that age, having children to support, and length of medical practice were significantly different among the shifters and non-shifters. These variables played an important role in the physician's intention to shift to a nursing career. A study has shown that relationships are fundamental components of "physician job satisfaction" and that different aspects of the satisfaction have differing relevance to specific physician subgroups [12]. In the present study, the findings that the demographic profile of shifters tended to be older, had children to provide for, and had been in practice longer than non-shifters suggested a fundamental difference between the two groups in terms of life experience and family responsibilities.

It should be noted that this finding of shifters as being relatively older differs from previous studies -- that of an inverse relationship between age and job mobility [13,14]. Also, Stamps *et al*. proposed that the level of satisfaction generally increased with the length of time spent in a job [15]. Given the long years of training necessary to attain the skills and knowledge for a medical specialist, it follows that age and length of medical training are directly related. Thus an interesting question in the study arises as to why older and more established doctors are shifting to a career in nursing compared to younger doctors?

Taking these results in the context of the medical profession, we see that these factors are interrelated. A British study made among young doctors to determine what was attractive in their careers found two aspects: *first*, vocational (clinical freedom, team work, varied clinical tasks, continuity of care); and *second*, the maintenance of the personal-professional boundaries (opportunity for flexible working, control over working pattern, personal time, accommodation of family life). The factors contributing to dissatisfaction was distaste for management and concern about professional isolation [16]. The fact that these unattractive aspects of work became more pervasive among older doctors might explain the findings in this study.

Also Stamps *et al*. proposed that the level of satisfaction generally increases with the length of time spent in a job [15]. Given the long years of study and economic cost of training invested in a medical career, doctors have certain goals and expectations in their profession which when not met after years of medical practice could lead to intense levels job dissatisfaction. How is this relevant to the findings of the study? As noted in the data, those who shifted careers were doctors who were in practice longer. The more specialized and more established medical practitioners were the ones leaving the practice. Many respondents mentioned that the stress and demands of the profession (e.g. heavy workload, long working hours) combined with the lack of opportunity for further growth, both, at the personal and professional level was a situation that many doctors faced. Moreover, all of this was in the context of a neglected health sector in the country with an increasingly hostile environment towards medical practice with the upswing of malpractice litigation.

So how does shifting to a nursing career overseas then address issues on career advancement and growth among doctors, when the shift is actually a downward movement career-wise? Alderfer's Existence-Relatedness-Growth (ERG) theory, a modification of Maslow's hierarchy of needs provides a clue to understanding this paradox [11]. The ERG theory contains a frustration-regression dimension wherein it is asserted that if the gratification of a higher-level need is stifled, the desire to satisfy a lower level need correspondingly increases. In the case of our medical doctors then, the unmet growth needs has created an impetus to fulfill lower level needs such as "existence needs" (i.e. salary/compensation). Since these doctors feel unable to substantially change their present career situations, it is a valid response to focus more on meeting other needs in the context of their family. Frustration then with the present career leads to regression to a lower-level need.

#### Level and sources of satisfaction

Can we relate this greater focus on *existential needs *among medical doctors to career shift intentions? How did this regression to lower level needs lead to career shift intentions? Herzberg's Two-Factor theory allows us to look separately at factors that cause satisfaction as distinct from factors that cause dissatisfaction [11,17]. Herzberg's theory asserts that sources of satisfaction are drawn from factors *intrinsic *to the job while sources of dissatisfaction are drawn from factors *extrinsic *to the job. Factors that contribute to satisfaction are different from factors that cause dissatisfaction. It follows then that those who are both less satisfied and more dissatisfied with their jobs are more vulnerable to shift careers. Looking at the specific factors which contributed to the level of satisfaction and dissatisfaction, across the board in this study, shifters tended to be less satisfied in terms of motivation factors and more dissatisfied in terms of hygiene factors as compared to non-shifters.

Doctors who were less satisfied with their jobs identified what is described by the ERG theory as "growth needs" as sources for lack of satisfaction. Specifically, the respondents pointed to unmet needs for career advancement, career growth, advance training, development of new skills, and personal accomplishment. With these unmet needs, the assertion of the ERG theory -- that unmet higher-level needs drive one to focus more on lower-level needs -- would explain why shifters prioritized needs such as higher compensation/income, availability of medical resources, better health policies/laws, and overall job security.

This was in contrast to non-shifters who were more satisfied in terms of motivation factors and less dissatisfied in terms of hygiene factors as compared to shifters. Having practiced medicine for a shorter period than shifters, the non-shifters had not reached the point yet where they felt that their gratification of higher level needs (i.e. career growth and advancement) was stifled; regression to satisfy a lower level need was not that urgent or strong for them so as to shift careers. The findings that non-shifters tended to be younger and a greater number having no children, indicates that these factors had a bearing on the choice not to shift to another career offering greater returns in terms of material compensation and benefits.

A closer look at the data suggests that taken in the context of the medical profession, it is the level of job satisfaction or dissatisfaction that is the immediate antecedent in the intention to shift careers among medical doctors. Additionally, socio-demographic factors, specifically age, having children to support, and length of medical practice gain explanatory power when they are linked to job satisfaction and dissatisfaction.

### Non-differential Variables in Physician Career Shift

#### Perceived benefits and costs of shifting to a nursing career

The findings in the study suggest no difference in the perceived benefits and cost of shifting to a nursing career among both shifters and non-shifters. This suggests a lack of direct influence on career shift intentions. So the question as to why many doctors decide to shift careers at this point in time, suggests that there is more to the career shift phenomenon than just one's perceptions about the alternative job.

#### Social network/Linkages

Social networks and linkages do not appear to be different among career shifters and non-shifters. Both shifters and non-shifters alike were noted to have linkages with other doctors who shifted to a nursing career abroad. In the study, both groups were noted to have a network of people who played a major role in this important career decision. The central role of the family as an institution was evident in the data. A majority of the shifters indicated that their nuclear and extended families exerted a heavy influence on their intention to shift to a nursing career. Although collegial networks came in a close second in the degree of influence, the preponderance of their social linkages pointed to familial relationships and friendship ties. Although literature is abundant on the role of informal networks of colleagues facilitating and opening doors for career advancement, the ultimate arbiter of career shift decisions lies in the hands of the immediate family [18,19]. It is not just the opportunity for career movement that influences the career-shift intention but, more important, the support of the spouse and parents. The implication is evident that if work opportunities present an incentive for a better family life it will be a strong impetus for career shift intentions.

Looking at the specific phenomenon in the Philippines, the major destination country for the majority of our shifters is the United States which notably offers immigration opportunity for the whole family. It specifically targets health workers with strong family ties removing a potential hindrance for immigration. This condition, in fact, facilitates the decision to shift careers by including the family as a unit for recruitment.

As with shifters, the non-shifters in this study, perceived their social networks not as a hindrance to career shift intentions but as supportive. Familial ties also played a major role in the career decisions. Parental influence was heavy among non-shifters reflective of the social status of many of the non-shifters, *i.e*. younger medical practitioners. What was surprising was that a greater proportion of non-shifters perceived their social network as supportive in the eventuality that they decided to change jobs. Although a majority of them would not consider shifting to a career in nursing in the near future, those who indicated that they might consider such a career move included economic and familial considerations as possible reasons.

## Conclusion

What then are we to make of this career shift phenomenon among medical doctors in Tacloban City? Data from the study indicates that medical doctors who have opted to shift careers to work overseas as nurses are not driven simply by economic motivations. Although financial consideration, admittedly, has played an important part in the career shift process it has not been the only influencing factor. Various other factors have influenced the decision to abandon their practice as medicine doctors -- albeit temporarily for some. Migration opportunities, career frustrations, poor working and socio-political environment are some of the other factors identified. On the other hand, those who have decided to remain in the medical profession have pointed to satisfaction with their present careers as their primary motivation. Other factors have included family unity and cost of retraining. It is apparent that for both shifters and non-shifters working as a nurse abroad is a tempting alternative to their present profession.

What ties together the various factors that have made medical doctors shift careers? The desire to emigrate to the U.S.A. or other countries is not a new experience for Filipinos. Various political and economic studies have theorized about the role of our colonial history in the shaping of social consciousness and the perception of emigration as a symbol of success or its equivalent. Thus opportunities to migrate, especially to the United States are welcomed with open arms. In the past decades, the increased demand for health workers -- particularly nurses -- in the international labor market opened doors for medical professionals to enter the United States with immigrant visas. What has resonated in the study is the sense of frustration with their careers along with the failing healthcare system of the country. The interplay of these factors has pushed medical doctors out of their professions and pulled them into careers as nurses working abroad.

What, then, differentiates shifters from non-shifters?

The data analysis above has shown several important points.

*First*, a career shift intention is influenced by one's satisfaction or dissatisfaction with the profession in that the less satisfied a person is with intrinsic aspects of the job and the more dissatisfied a person is with the extrinsic aspects of the job, the higher the tendency to shift to another career. In the context of the medical doctors, personal factors such as age, length of practice, and children to support, influence the level of job satisfaction or dissatisfaction in that the older one gets and the longer one stays in a job, the higher the level of dissatisfaction and lesser is the degree of satisfaction.

*Second*, applying Alderfer's ERG theory and Herzberg's Two-Factor theory can provide a useful theoretical basis for understanding the career shift phenomenon among medical doctors. The ERG theory through its frustration-regression component explains why existence needs such as salary/compensation has become more important than higher level needs such as career growth and advancement, for the job shifters. The practical implications of Herzberg's theory provide a useful framework for analysis of the levels of job satisfaction and dissatisfaction.

*Third*, data from the study also suggests that the perception about opportunity costs of shifting to an alternative career, was not a differential factor in career shift intentions among the doctors in the study. Though many doctors become nurses to enjoy the benefits of working abroad, the shift actually provides an avenue for getting out of a career that has become less satisfying over years of medical practice. It does not directly influence career shift intentions independently.

*Fourth*, the role of social networks -- found to be present among both shifters and non-shifters -- did not prove to be a hindrance to career shift intentions. For both groups in the study, almost all their important relationships in terms of career decisions were supportive of intention to change jobs.

Finally, two major issues were identified among medical doctors with intentions to shift careers -- the lack of opportunity for professional growth and the lack of adequate compensation and benefits for their work at home.

The study suggests that for a local community like Tacloban City to retain medical doctors, it is not prudent to focus mainly on monetary compensation. As important is the need to address non-monetary factors that determine the physician's level of job satisfaction or dissatisfaction. These include, but are not limited to, creating more opportunities for professional growth, appropriate recognition for work, avenues to acquire advance training, increase the value of work done by physicians, improve health policies and laws, overall job security and increase resources available to support work. Since this is an exploratory study limited to a particular locale in the central region of the country, it is further recommended that similar studies be done in other parts of the Philippines to better understand this phenomenon at a national level.

## Abbreviations used

CGFNS: Commission on Graduates of Foreign Nursing Schools; NCLEX: National Council Licensure Examination; RHUs: Rural Health Units; USA/United States: United States of America; WHO: World Health Organization.

## Competing interests

The author declares that they have no competing interests.

## Authors' contributions

MPL started work on this research for her graduate degree program thesis and subsequently edited it for publication.

## Author's information

Meredith del Pilar-Labarda is currently the Chair of the Medical Department, School of Health Sciences, University of the Philippines Manila, Palo, Leyte campus
